# Gut microbiota’s role in glioblastoma risk, with a focus on the mediating role of metabolites

**DOI:** 10.3389/fneur.2024.1386885

**Published:** 2024-07-03

**Authors:** Junqing Yan, Bo Li, Chun Luo

**Affiliations:** ^1^Nanxiang Branch of Ruijin Hospital, Shanghai, China; ^2^Tongji Hospital Affiliated to Tongji University, School of Medicine, Tongji University, Shanghai, China

**Keywords:** Mendelian randomization, glioblastoma, gut microbiota, metabolites, brain-gut axis

## Abstract

This study employed Mendelian randomization (MR) analysis to systematically investigate the potential connections between gut microbiota and the risk of glioblastoma (GBM). We identified 12 microbial groups closely associated with the incidence risk of GBM. Subsequently, MR analysis was conducted on 1,091 blood metabolites and 309 metabolite ratios, revealing 19 metabolites that exert an impact on the occurrence of GBM. Hypothesizing that gut microbiota may influence the risk of glioblastoma multiforme by modulating these metabolites, we performed MR analyses, considering each microbial group as exposure and each metabolite as an outcome. Through these analyses, we constructed a regulatory network encompassing gut microbiota, metabolites, and GBM, providing a novel perspective for a deeper understanding of the role of the gut-brain axis in the pathogenesis of GBM. This research offers crucial insights into how gut microbiota may affect the risk of GBM by regulating specific metabolites. The identified regulatory network of the gut-brain axis may play a significant role in the formation and development of GBM, providing valuable information for future research and therapeutic interventions.

## Introduction

Glioblastoma (GBM), the most malignant primary brain tumor within the central nervous system, represents a formidable challenge in neuro-oncology (1). Current standard treatment modalities include surgery, radiotherapy, and temozolomide chemotherapy (2). Despite the implementation of these interventions, the prognosis for GBM patients remains bleak, and the underlying pathogenic mechanisms of the disease remain incompletely understood.

In recent years, research on the gut microbiota has garnered widespread attention, encompassing various physiological processes, including immune regulation, nutritional metabolism, and the generation of bioactive molecules. The associations with a variety of diseases are gradually coming to light (3–5). Meanwhile, there exists a bidirectional signaling pathway between the gut and the brain, known as the gut-brain axis. This axis involves various pathways, including neural, endocrine, and immune signaling, allowing the gut microbiota to influence the central nervous system (6–8). Researches have already identified a potential association between alterations in the composition of the gut microbiota and the risk of GBM (6, 9). A recent Mendelian randomization (MR) study has also identified several gut microbiota species that have a causal relationship with GBM. However, the precise mechanisms through which the gut microbiota exerts its influence on GBM have not been fully elucidated (10).

Metabolites play a pivotal role in various physiological functions, and the intricate interplay between metabolic pathways and the biology of GBM has emerged as a focal point of research. Metabolites derived from both endogenous cellular processes and exogenous sources (including diet and the gut microbiota) can participate in GBM processes such as proliferation, apoptosis, and angiogenesis through diverse pathways. This includes influencing dysregulated signaling pathways within GBM cells, modulating the tumor microenvironment, and impacting immune responses (11–13).

Nevertheless, there is currently a lack of systematic studies exploring which metabolites can control the risk of GBM. A recent study has provided convenient Genome-Wide Association Study (GWAS) data for 1,091 blood metabolites and 309 metabolite ratios, offering a valuable resource for addressing this research gap (14).

## Method

### Data sources

The gut microbiota data were sourced from Mibiogen.^1^ After excluding unidentified microbial taxa, GWAS data for 196 gut microbiota species were obtained, spanning 9 Phyla, 16 Class, 20 Order, 32 Family, and 119 Genus. Metabolite GWAS data were derived from the latest GWAS study, encompassing data for 1,091 blood metabolites and 309 metabolite ratios, (PMID36635386) (14). GWAS data for GBM were obtained from Finngen (finn-b-C3_GBM_EXALLC), comprising 91 patient cases and 174,006 control subjects, all of European descent.

### Mendelian randomization design

#### Selection of instrumental variables (IVs)

The selection of IVs is based on the three requirements of MR: (1) Strong Correlation with the Exposure Factor: Single nucleotide polymorphism (SNPs) chosen as IVs need to exhibit a strong correlation with the exposure factor. The standard requirement is typically set at *p*-value <5e-8, while in instances where an insufficient number of IVs meet this criterion, it may be relaxed to *p* < 1e-5. Specific criteria will be outlined in each MR analysis. To emphasize the strength of correlation, a requirement is imposed that the *F*-value be greater than or equal to 10. The formula for calculating *F* is as follows (15, 16):


$$F=\left(N-2\right)*R^{2}/\left(1-R^{2}\right)$$

$$R^{2}=\left(2*\mathrm{bet}a^{2}\right)/\left(2*\mathrm{bet}a^{2}+2*N*se^{2}\right)$$

*N* represents the sample size of the exposure data. (2) Independence of IVs: To ensure the independence of IVs, when the data are considered as the exposure factor, a requirement is set for the linkage disequilibrium coefficient (*r*^2^) to be less than or equal to 0.001, with a region width of 10,000 kb. This standard is applicable to each subsequent MR analysis (17, 18). (3) Not Regulated by Other Confounding Factors for the Outcome: For this purpose, each remaining SNP was individually scrutinized through the Phenoscanner website^2^ to exclude associations with any confounding factors potentially linked to the outcome.

#### Analysis methods

In each step of the MR analysis, three methods were employed: inverse variance weighting (IVW), MR-Egger regression, and the weighted median estimator (WME).

IVW is a widely used method that involves weighting the effect sizes of candidate SNPs by the inverse of their variances. This method assumes that all SNPs are valid IVs, and their weights are inversely proportional to their variances (19). MR-Egger regression estimates the average pleiotropy through an intercept, even when all IVs cannot completely eliminate pleiotropy. It provides a test for non-zero average pleiotropy and can be used to detect potential horizontal pleiotropy (20). WME estimates the causal effect through the median, making it robust to up to 50% of IVs being subject to pleiotropy (20). Which method dominates the final result depends on data heterogeneity and pleiotropy. If neither heterogeneity nor pleiotropy is present, the IVW method’s estimates are preferred. If heterogeneity exists without pleiotropy, the Weighted Median method’s results are favored. If pleiotropy is detected, the MR-Egger method’s results take precedence (20).

Based on the heterogeneity and pleiotropy test, primary results are based on IVW, with MR-Egger and WME serving as supplementary analyses, providing additional insights when necessary.

#### Heterogeneity, pleiotropy, and sensitivity tests

Heterogeneity among IVs’ estimates was assessed to examine the consistency of causal effects across different genetic variants. The Cochran *Q* test was employed, where a significant *Q* statistic suggests heterogeneity. MR Pleiotropy and Horizontal Pleiotropy and Mendelian Randomization-Pleiotropy Residual Sum and Outlier (MR-PRESSO) are used to evaluate genetic pleiotropy and refine estimates by identifying and eliminating outliers (21). Sensitivity analyses were conducted to evaluate the robustness of results by excluding outliers or influential genetic variants by “leave-one-out” method. Results demonstrating significant pleiotropy and heterogeneity (*p* < 0.05) were systematically excluded from the analysis.

In accordance with the aforementioned criteria, we conducted separate MR analyses for the gut microbiota and GBM, as well as for metabolites and GBM. Subsequently, individual MR analyses were performed for the selected gut microbiota and metabolites identified as being associated with the risk of GBM.

### Evaluation of mediating effect

**Figure 1:**
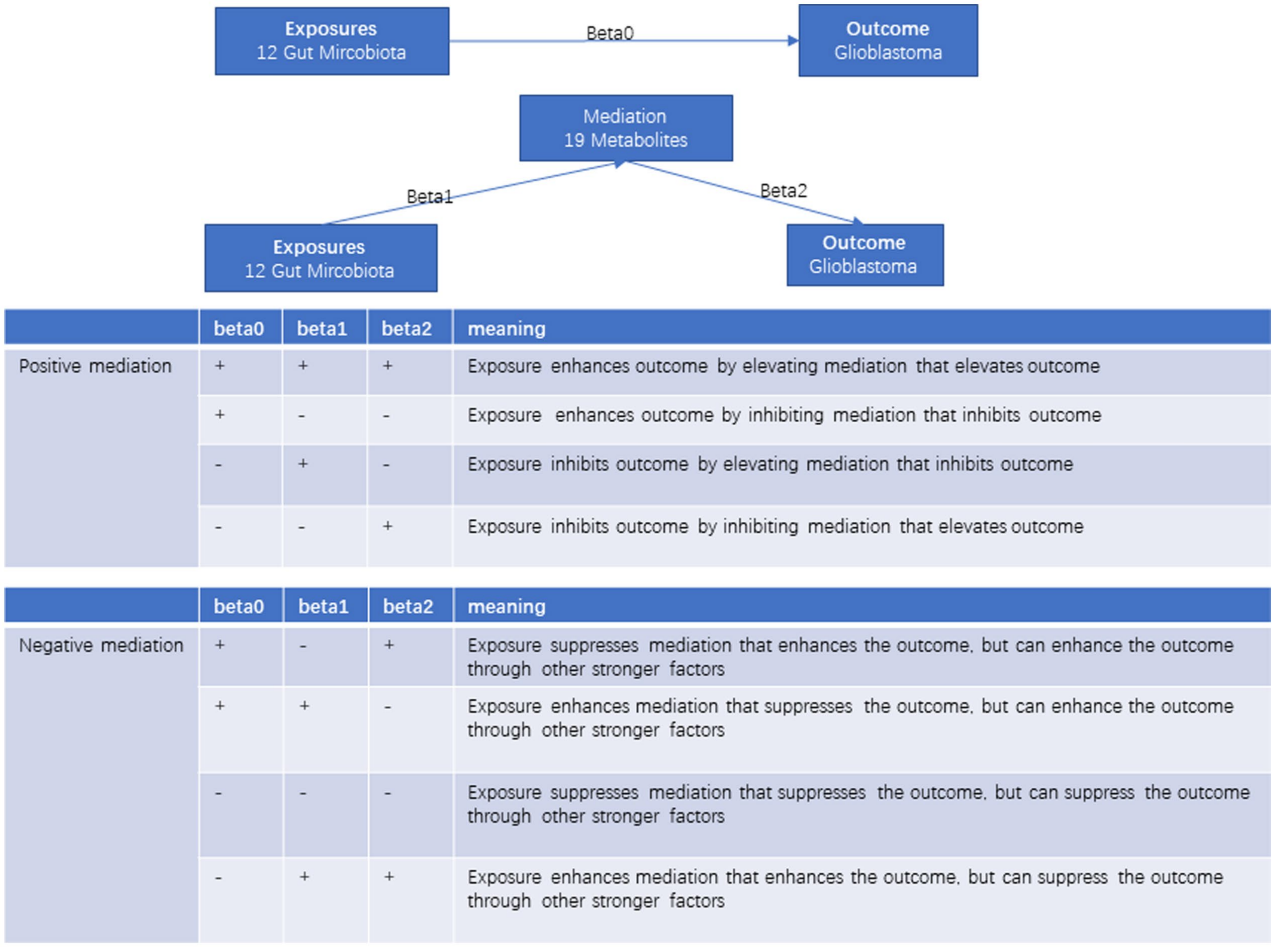
The diagram of mediating effects.

In the two-step MR analyses, the calculation of the mediation effect is as follows: the effect of the microbiota on GBM is denoted as beta0, the effect of the microbiota on the metabolite is denoted as beta1, and the effect of the metabolite on GBM is denoted as beta2. The mediation effect (b) is computed as beta1*beta2, and the direct effect is beta0 – b. In statistics research, the mediation effect explains how a variable influences a dependent variable. This mechanism typically involves a mediating variable. Mediation effects can be positive, enhancing the relationship, or negative, diminishing it. Positive mediation strengthens the effect of the independent variable on the dependent variable, while negative mediation weakens or nullifies this effect (Figure 1). As the gut microbiota can regulate GBM not only through metabolites but also through other mechanisms, this mediation effect is considered incomplete and, in some cases, may even be opposite to the direct effect. In such situations, the metabolite is considered a negative mediator. Finally, we visually represented the regulatory network of the gut microbiota-metabolite-GBM through a Sankey diagram.

## Results

### MR analysis for gut microbiota and GBM

A total of 196 gut microbiota species were considered as exposures, with each microbiota having SNPs with *p*-values less than 1e-5 selected as IVs. GBM served as the outcome, and MR analyses were separately conducted for each microbiota. Results deemed meaningful were those with *p*-values less than 0.05 in the IVW method. After excluding results exhibiting heterogeneity and pleiotropy, a total of 12 microbiota species were identified as associated with GBM.

**Figure 2:**
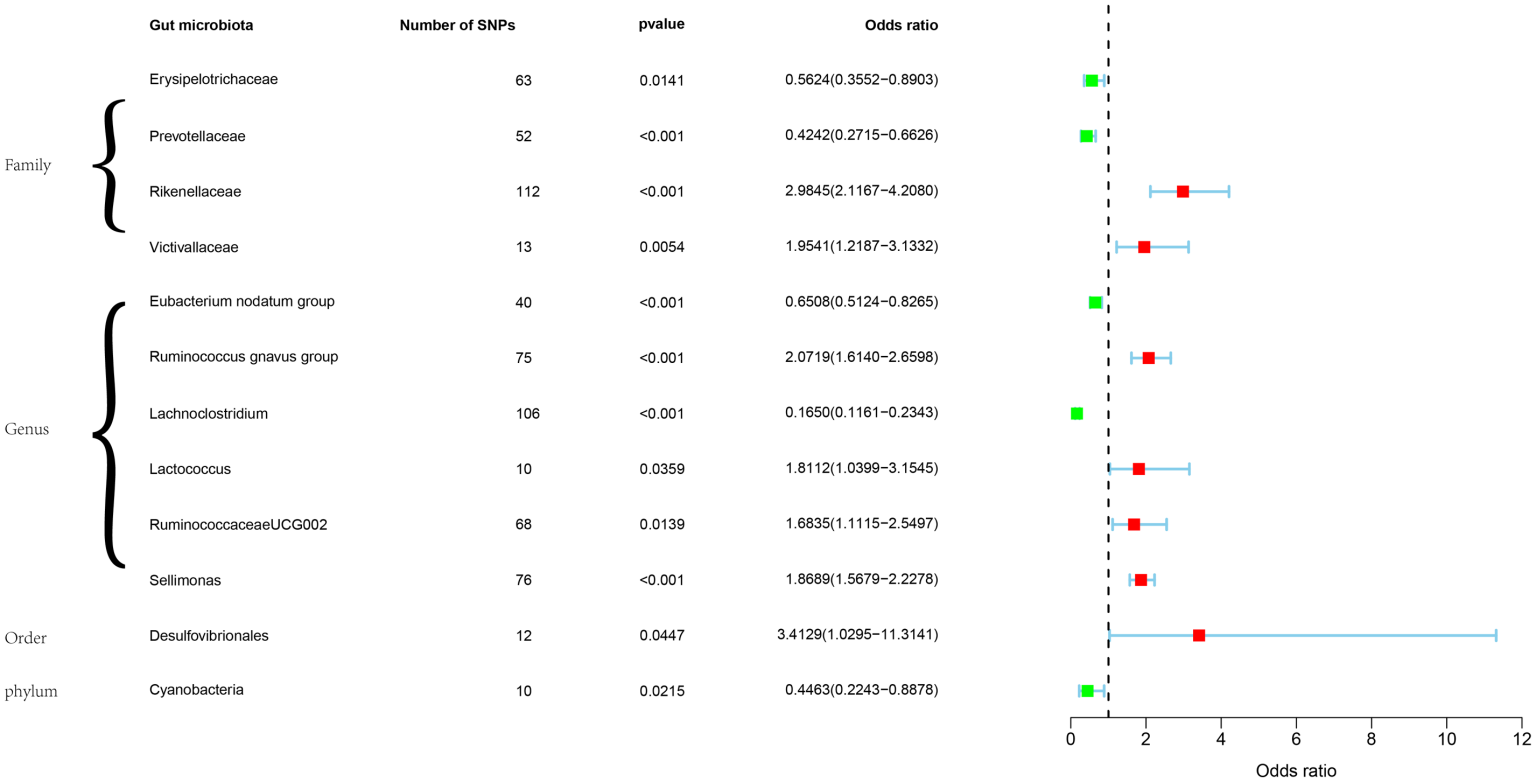
Forest plot of the associations between 12 bacterial traits with the risk of GBM.

Among these, families of *Erysipelotrichaceae*, *Prevotellaceae*, genuses of *Eubacterium nodatum* group, *Lachnoclostridium*, and phylum of *Cyanobacteria* were identified as protective factors against GBM. Conversely, families of *Rikenellaceae*, *Victivallaceae*, *Ruminococcus gnavus* group, *Lactococcus*, *Ruminococcaceae* UCG002, *Sellimonas*, and order of *Desulfovibrionales* were associated with an increased risk of GBM (Figure 2).

The SNPs used as IVs in this step were shown in Supplementary Table S1; The detailed MR results can be found in Supplementary Table S2; The heterogeneity and pleiotropy test results were illustrated in Supplementary Table S3; Result of sensitivity analyses by “leave-one-out” method were presented in Supplementary Figure S1.

We further performed reverse Mendelian randomization and found reverse causality between genus *Lactococcus* and GBM (IVW, *p* = 0.018, OR = 1.070, 95%CI = 1.012–1.133), indicated that the occurrence of GBM can change the composition of this genus. Meanwhile, the effect of other microflora on GBM is unidirectional.

### MR analysis for metabolites and GBM

A total of 1,091 blood metabolites and 309 metabolite ratios were considered as exposures, with SNPs for each metabolite having *p*-values less than 5e-8 selected as IVs. GBM served as the outcome in the MR analysis. Results considered meaningful were those with *p*-values less than 0.05 in the IVW method and lacking heterogeneity and pleiotropy. A total of 19 metabolites were identified as associated with GBM.

**Figure 3:**
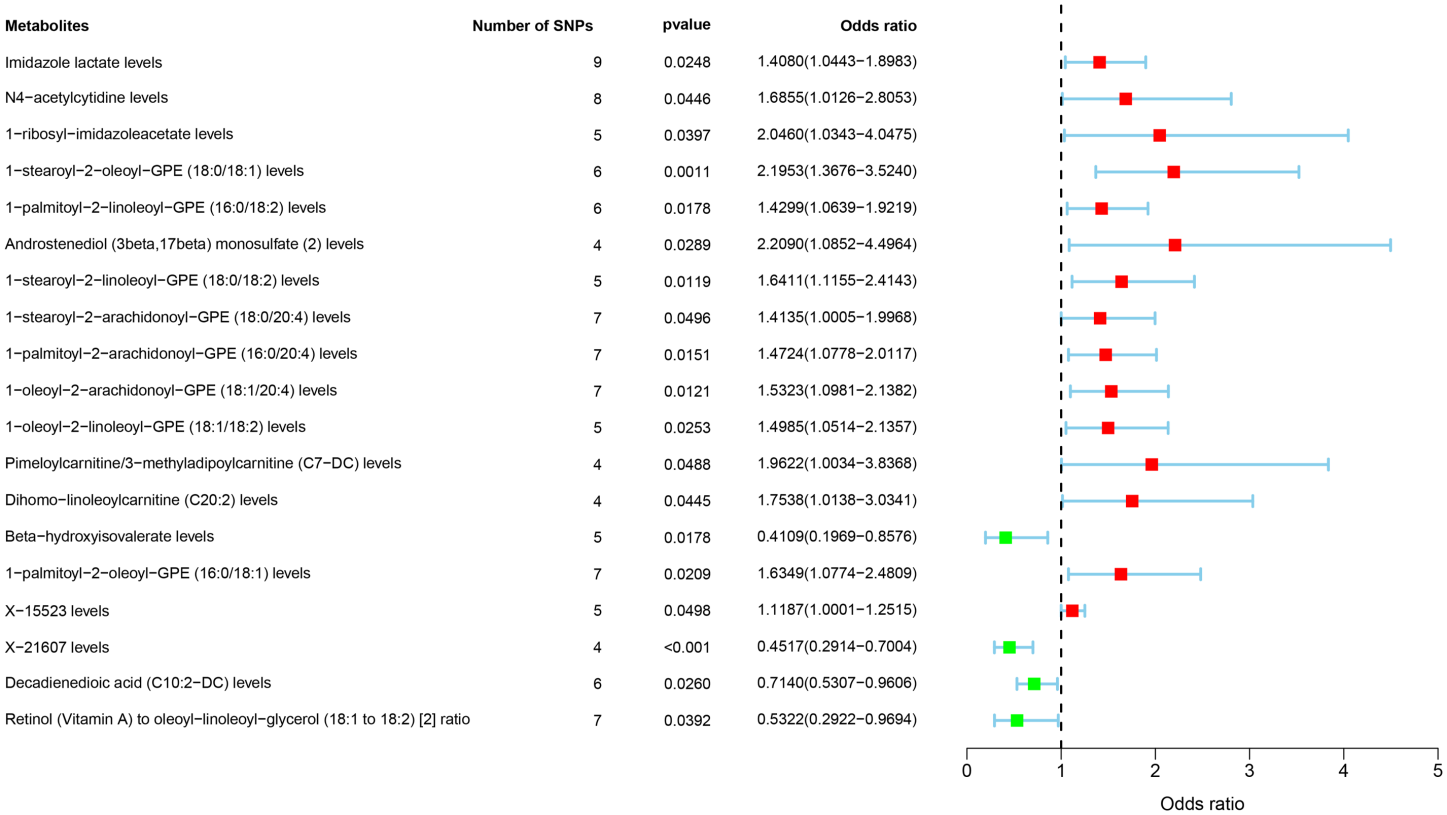
Forest plot of the associations between 19 metabolites traits with the risk of GBM.

Metabolites such as Imidazole lactate, N4-acetylcytidine, 1-ribosyl-imidazoleacetate, 1-stearoyl-2-oleoyl-GPE (18:0/18:1), 1-palmitoyl-2-linoleoyl-GPE (16:0/18:2), Androstenediol (3beta,17beta) monosulfate (2), 1-stearoyl-2-linoleoyl-GPE (18:0/18:2), 1-stearoyl-2-arachidonoyl-GPE (18:0/20:4), 1-palmitoyl-2-arachidonoyl-GPE (16:0/20:4), 1-oleoyl-2-arachidonoyl-GPE (18:1/20:4), 1-oleoyl-2-linoleoyl-GPE (18:1/18:2), Pimeloylcarnitine/3-methyladipoylcarnitine (C7-DC), Dihomo-linoleoylcarnitine (C20:2), 1-palmitoyl-2-oleoyl-GPE (16:0/18:1), X-15523 were associated with an increased risk of GBM. Conversely, Beta-hydroxyisovalerate, 1-palmitoyl-2-oleoyl-GPE (16:0/18:1), X-21607, Decadienedioic acid (C10:2-DC), Retinol (Vitamin A) to oleoyl-linoleoyl-glycerol (18:1–18:2) (2) ratio were associated with a decreased risk of GBM (Figure 3).

The MR results can be found in Supplementary Table S4, The SNPs used as IVs was showed in Supplementary Table S5; The heterogeneity and pleiotropy test results can be found in Supplementary Table S6; Result of sensitivity analyses by “leave-one-out” method were illustrated in Supplementary Figure S2.

Reverse MR Analysis showed that only Pimeloylcarnitine/3-methyladipoylcarnitine (C7-DC) was significant (IVW, *p* = 0.003, OR = 0.957, 95%CI = 0.930–0.985), indicating that it was regulated by the occurrence and development of GBM, while the rest metabolites all had a one-way causal relationship with GBM.

### MR for microbiota-metabolite interactions

**Figure 4:**
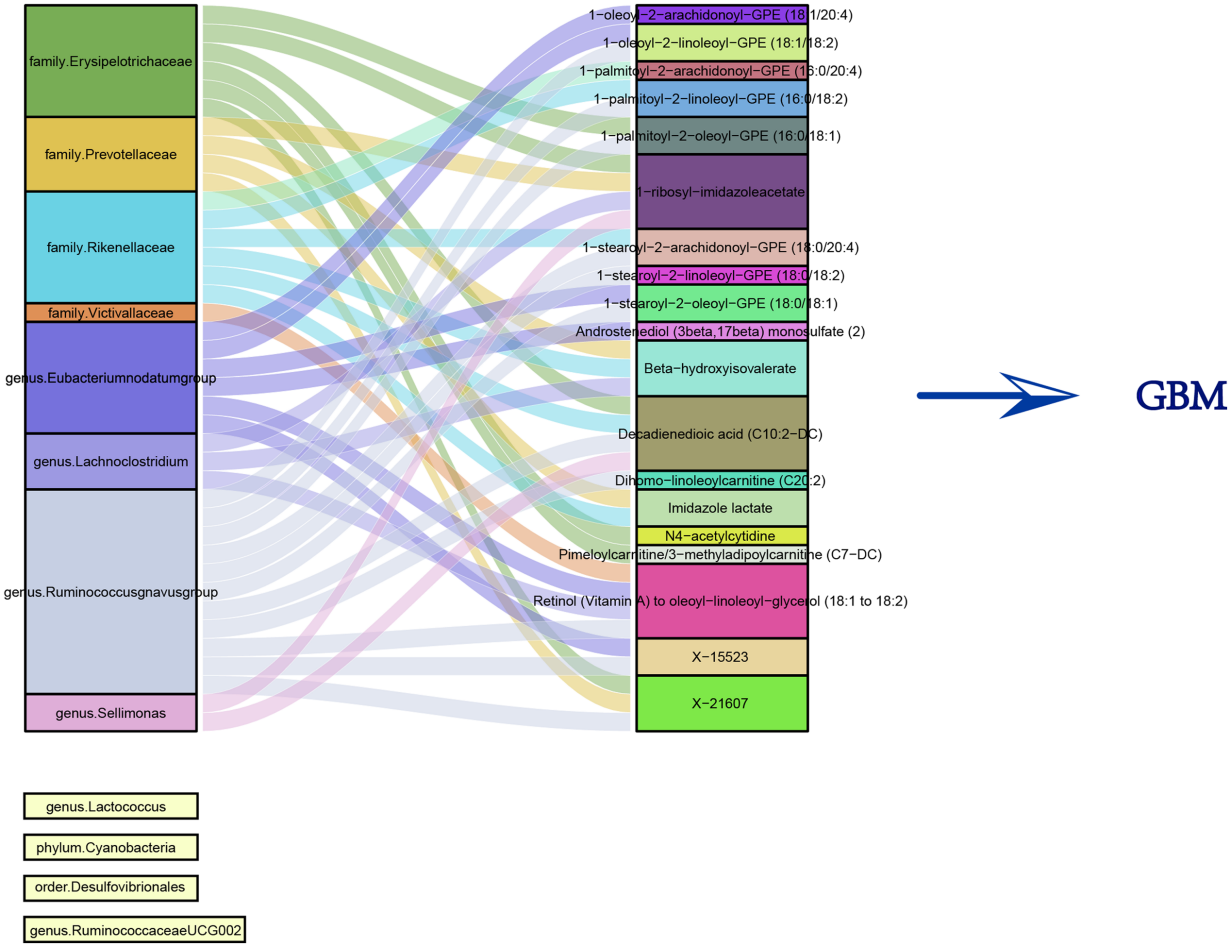
Sankey diagram of the correlation between 12 gut microbiota and 19 metabolites.

Utilizing the 12 microbiota species identified earlier, each microbiota with SNPs having *p*-values <1e-5 as IVs, and considering the 19 metabolites as outcomes, multiple MR analyses were conducted. The results revealed that eight microbiota species had a significant impact on the metabolites. Meanwhile, the regulation of GBM by the remaining four microbiota species was found to be independent of the influence on metabolites. Figure 4 illustrated the relationship between these gut microbiota and metabolites.

We summarized the specific MR Analysis results of these flora and metabolites, the IVs used, and the results of heterogeneity and pleiotropy tests and presented them in Supplementary Tables S7–S9.

### Assessment of mediation effects

We separately calculated the mediation effects of gut microbiota on GBM risk through metabolites, categorizing these effects into positive and negative mediations, and illustrated them in Supplementary Table S10.

## Discussion

MR study stands out as a robust method for causal inference, leveraging the natural distribution of genetic variants to emulate randomized experiments. Its distinctive strengths include the mitigation of confounding factors prevalent in observational studies, the avoidance of selection bias due to the predetermined nature of genetic variation, and enhanced translatability of results to clinical applications.

There are several previous MR studies focusing on the impact of gut microbiota in GBM. Wang et al. revealed a positive correlation between GBM risk and family of *Peptostreptococcaceae* and genus of *Eubacterium brachy* group and a negative correlation with the family of *Ruminococcaceae*, and genus of *Anaerostipes*, *Faecalibacterium*, *Lachnospiraceae* UCG004, *Phascolarctobacterium*, *Prevotella 7*, *Streptococcus* (22). In addition, the protective effect of family *Ruminococcaceae* and the harmful effects of family *Bacteroidaceae* and *Peptococcaceae* and genus *Eubacterium* (brachy group), *Actinomyces*, *Bacteroides* and *Ruminiclostridium 6* for GBM were illustrated by Ju et al. (23). In our study, utilizing the same exposure and outcome, MR analysis was conducted, but discrepancies in results were observed, potentially attributed to variations in data processing and statistical methods, of which the most significant difference was the multistep way of choosing SNPs.

Besides MR methods, researchers also using Metagenomic PCR-DGGE, Illumina-based Hiseq 2500 Highthrough-put sequencing, and real-time PCR to explore the impact of gut microbiota on GBM (24). However, they only found the difference of microflora between patients and normal individuals without evidence of a causal relationship.

In comparison to the aforementioned studies, the highlight of our study is the MR analysis method with multistep approach to selecting SNPs. Additionally, a notable strength of our research lies in the integration of metabolomics, offering further exploration into the mediating role of metabolites in this context.

The presence of a brain-gut axis allows the gut and brain to interact. Gut microbes are able to regulate the function of various cells in the brain through a variety of metabolites they produce (25). Prior researches indicated that the gut microbiota may play a role in the occurrence, progression, and treatment of gliomas through metabolic regulation of the epigenetic and immune microenvironment. This effect can be achieved by altering the nervous system microenvironment or the epigenetics of tumor cells (9). These molecules play a crucial role in the initiation and progression of glioblastomas (26). Some explored mechanisms include the gut microbiota’s ability to inhibit SDF-1 through the production of certain metabolites, thus inhibiting the migration of glioma cells (27). Tryptophan metabolites activate the aryl hydrocarbon receptor (AHR) pathway, promoting tumor cell proliferation in gliomas, including astrocytomas, medulloblastomas, and glioblastomas (28). Imbalances in the gut microbiota leading to reduced concentrations of circulating short-chain fatty acids have been associated with disrupted morphology and function of microglial cells (29). This disruption, via stress-related pathways, influences tumor occurrence and prognosis. Butyrate affects the immune system by inducing Treg differentiation and modulating inflammation (30). Acetate and glucose collectively participate in the tricarboxylic acid (TCA) cycle, influencing the production of acetyl-coenzyme A, and participate in the apparent modification of isocitrate dehydrogenase (IDH), whose mutation is an important marker of glioma, thereby driving proliferation and survival (31). Moreover, in murine models, dysregulation of the gut microbiota downregulates the expression of Foxp3 in the brain and promotes the growth of gliomas (32). The gut microbiota can also regulate neurotransmitters, thereby playing a role in the onset and development of central nervous system diseases (33). On the therapeutic side, crosstalk of gut flora and blood–brain barrier can also alter the effectiveness of antitumor drugs in treating glioma (34).

A recent study has provided Genome-Wide Association Study (GWAS) data for 1,400 plasma metabolites, offering a robust foundation for exploring the roles of metabolites in diseases (14).

Based on these findings, the researchers are also working to explore what factors can regulate gut microbes and thus affect glioma progression. Here are some of the results that have worked so far: Bifidobacterium inhibits MEK/ERK cascade by altering the gut microbiome and improves the prognosis of glioma (35). Another possible stimulator of gut microbiota is high-glucose drink, which increase the content of *Desulfovibrionaceae* family in gut and inhibits the growth of GBM. The mechanism may be to induce changes in gene expression of CD8 T cells and thus affect the anti-tumor immune response (36). At the same time, edible fungi have also recently been identified as an important factor in altering the composition of gut microbes (37).

For the 12 gut microbiota species identified in this study, specific investigations revealing the impact of any individual species on GBM have not yet been identified. We hope that this study can provide novel insights for future researchers, further elucidating the mechanisms through which these gut microbiota may influence GBM.

Given these considerations, we systematically investigated the mediating role of metabolites in the gut microbiota on the GBM process. It is noteworthy that some of these mediating effects are negative, suggesting that certain gut microbial communities regulate not only metabolites but also other factors with more significant effects on GBM. This leads to an acknowledgment that the gut microbiota-metabolite-GBM network we constructed is incomplete. However, considering the complex signaling of the brain-gut axis, this incompleteness is understandable. We hope that future researchers can refine and enhance this network.

## Conclusion

Through multiple two-step MR analyses, this study preliminarily reveals the relationship between the gut microbiota and the risk of GBM, exploring potential mediating roles of metabolites. This discovery provides a new research direction for further investigating the pathogenic mechanisms of neurological tumors and establishes a theoretical foundation for the development of relevant therapeutic strategies.

## Data availability statement

The original contributions presented in the study are included in the article/Supplementary material, further inquiries can be directed to the corresponding author.

## Ethics statement

Ethical approval was not required for the study involving humans in accordance with the local legislation and institutional requirements. Written informed consent to participate in this study was not required from the participants or the participants’ legal guardians/next of kin in accordance with the national legislation and the institutional requirements.

## Author contributions

JY: Conceptualization, Data curation, Formal analysis, Funding acquisition, Investigation, Methodology, Project administration, Resources, Software, Visualization, Writing – original draft, Writing – review & editing. BL: Conceptualization, Data curation, Formal analysis, Funding acquisition, Investigation, Methodology, Project administration, Resources, Software, Visualization, Writing – original draft, Writing – review & editing. CL: Supervision, Validation, Writing – original draft, Writing – review & editing.

## Footnotes

^1^ https://mibiogen.gcc.rug.nl/

^2^ http://www.phenoscanner.medschl.cam.ac.uk/
