# Supplementary figures and images for: Gut microbiota’s role in glioblastoma risk, with a focus on the mediating role of metabolites
Source: Front Neurol. 2024 Jul 3;15:1386885. doi: 10.3389/fneur.2024.1386885 (PMC11253649; doi:10.3389/fneur.2024.1386885)

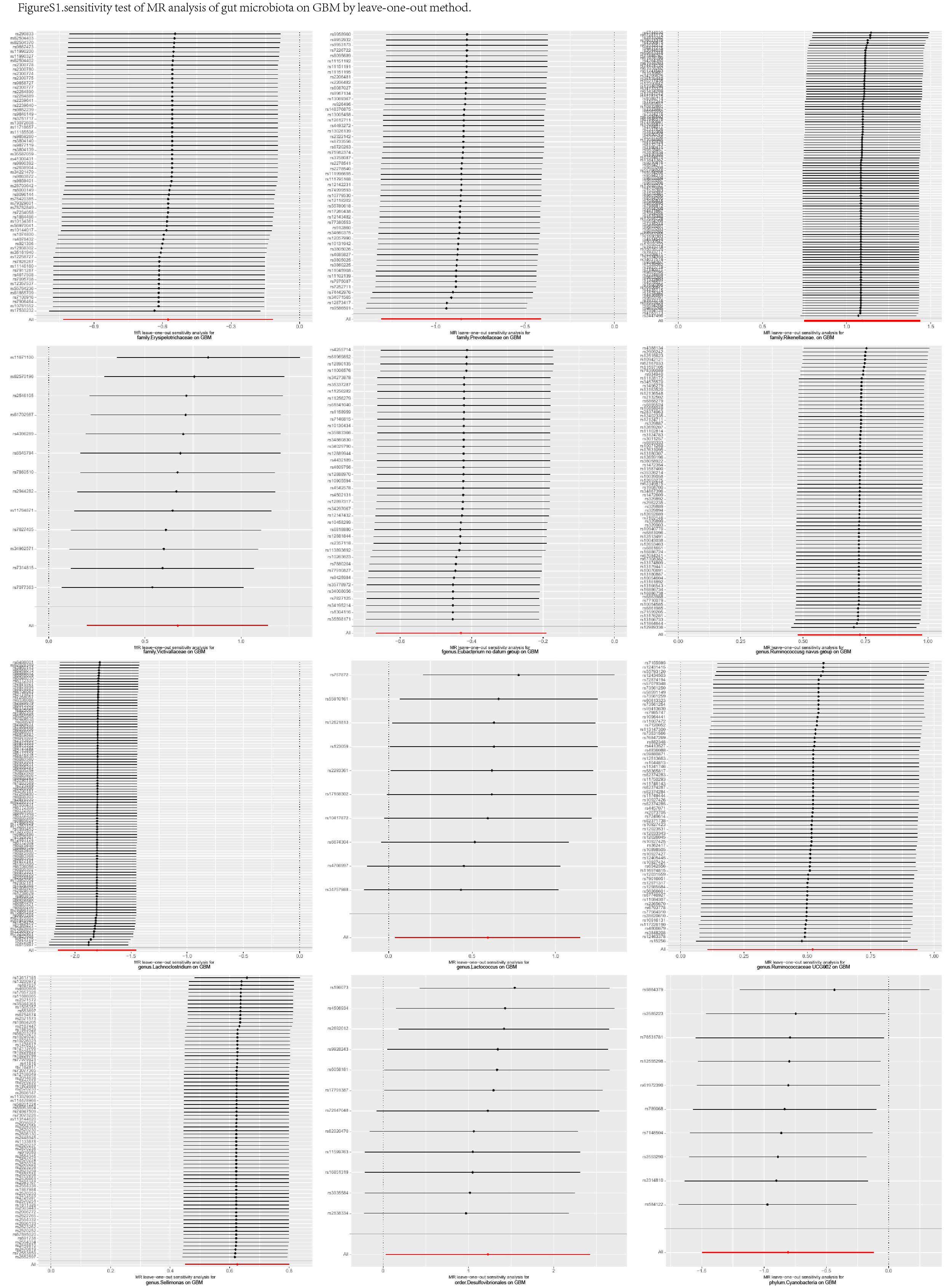

Supplement: Supplementary file 1 [file Image_1.TIF]
